# Caught in a Web of Necrosis

**DOI:** 10.1016/j.acepjo.2026.100432

**Published:** 2026-06-27

**Authors:** William Wanzor, Aakriti Bhargava

**Affiliations:** Department of Emergency Medicine, University Medical Center, Texas Tech University Health Sciences Center, Lubbock, Texas, USA

**Keywords:** brown recluse, dermonecrotic lesion, loxoscelism, necrotic arachnidism, pediatric, skin graft, skin necrosis, spider bite

## 1 Patient Presentation

**Figure 1:**
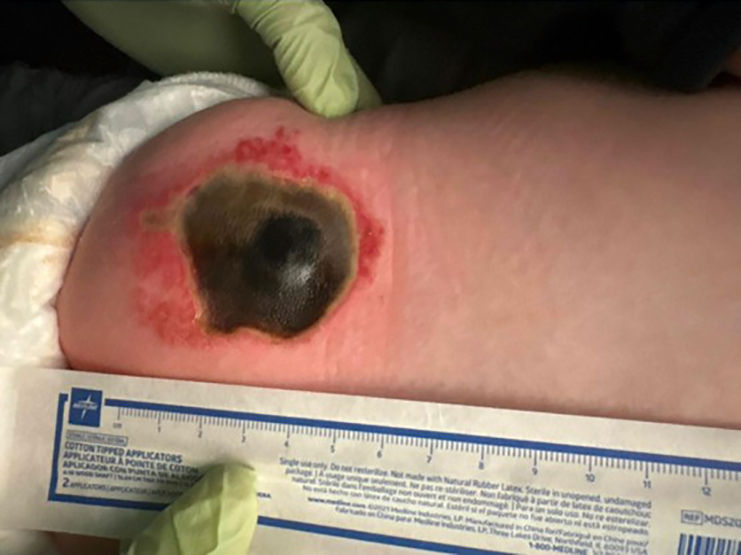
Initial appearance: dusky plaque with central black eschar on the left superior gluteal region.

**Figure 2:**
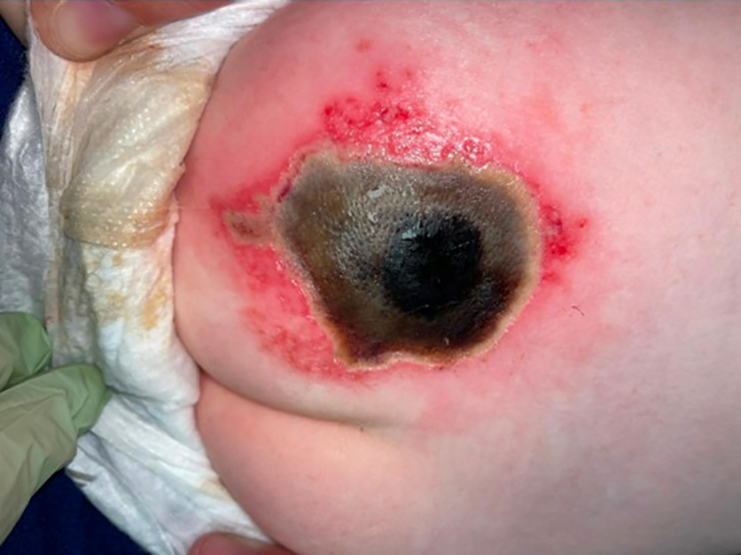
Measurement view: approximately 5 to 6 cm lesion diameter.

**Figure 3:**
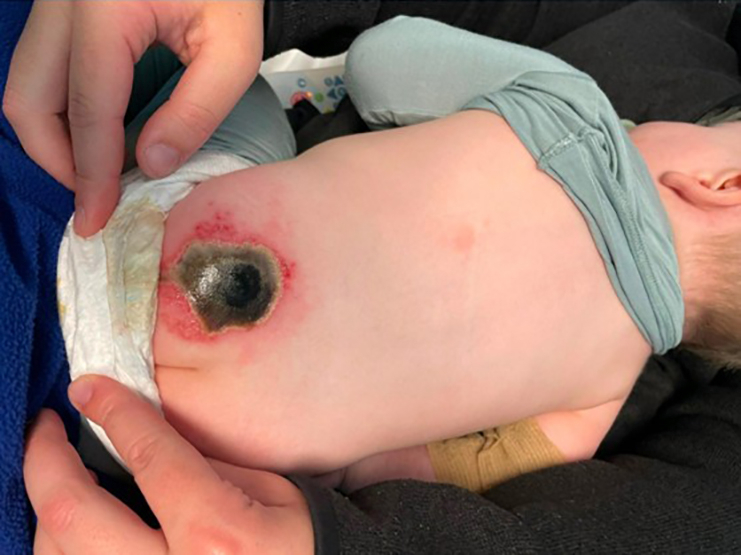
Alternate view with ruler: lesion size and surrounding erythema.

**Figure 4:**
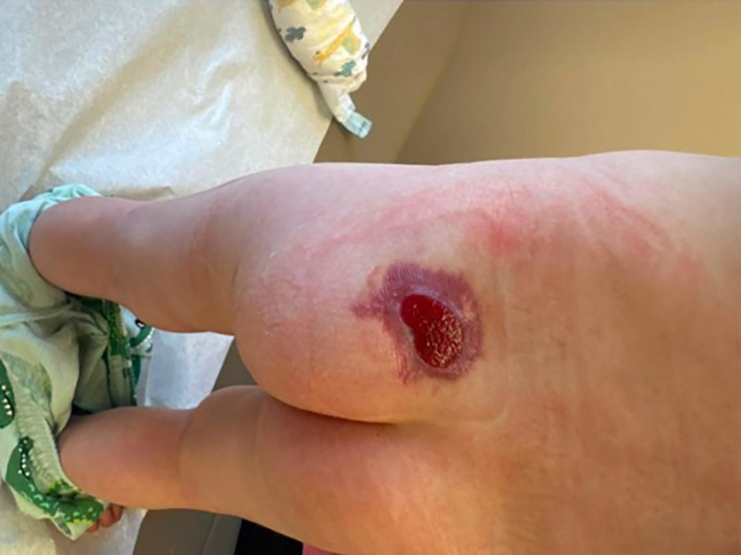
Follow-up: postdebridement/graft course with residual hypergranulation.

A previously healthy 15-month-old boy from West Texas presented with a rapidly progressive buttock lesion. Caregivers noted a dusky patch 2 days earlier that enlarged with blistering and a black center (Fig 1). A fall onto a ride-on toy the prior week raised concern for contusion; no injury or bite was witnessed. He appeared well and afebrile (temperature, 99.9°F) with a heart rate 155 bpm, blood pressure 118/59 mm Hg, Spo_2_ 97%, and a 5 × 6 cm dusky plaque with central eschar and surrounding erythema/maceration in the left superior gluteal region (Figure 1, Figure 2, Figure 3, Figure 4). White blood cell count was 11.5 × 10^9^/L with a normal coagulation profile. Tangential excision and debridement with biodegradable temporizing matrix placement were performed during hospitalization; split-thickness skin grafting followed weeks later. At 60-day follow-up, the residual wound measured 1.5 × 2.1 cm with hypergranulation treated with silver nitrate.

## 2 Diagnosis Presumed Loxoscelism (Brown Recluse–Associated Dermonecrosis)

The presumed loxoscelism diagnosis was based on characteristic cutaneous necrosis without another identifiable culprit in an endemic region. Cutaneous loxoscelism is most common, starting as erythema that can evolve into painful edema/induration over 12 to 24 hours, with necrosis evident by ∼72 hours. Less commonly, systemic disease may involve acute renal failure, intravascular hemolytic anemia, and thrombocytopenia. No medical therapy has proven efficacy; management is supportive wound care, with surgical debridement in severe cases. Only about one-third of moderate-to-severe cases have a documented spider bite, complicating diagnosis.^1^, ^2^, ^3^, ^4^

## Conflicts of Interest

All authors have affirmed they have no conflicts of interest to declare.
